# Impact on Prognosis of the Surgical Route, Laparoscopy or Laparotomy, for the Surgical Staging of Early Stage Ovarian Cancer—A Study from the FRANCOGYN Group

**DOI:** 10.3390/jcm9113528

**Published:** 2020-10-31

**Authors:** Margaux Merlier, Yohan Kerbage, Adeline Pierache, Nassima Ramdane, Geoffroy Canlorbe, Pierre-Adrien Bolze, Marcos Ballester, Sofiane Bendifallah, Lobna Ouldamer, Cyril Touboul, Cyrille Huchon, Vincent Lavoue, Yohann Dabi, Cherik Akladios, Charles Coutant, Emilie Raimond, Alexandre Bricou, Jerôme Phalippou, Pierre Collinet, Henri Azaïs

**Affiliations:** 1 Department of Gynecologic Surgery, Jeanne de Flandre Hospital, CHU de Lille, Avenue Eugène Avinée, 59120 Loos, France; mar.merlier@gmail.com (M.M.); yohan.kerbage@gmail.com (Y.K.); Jerome.PHALIPPOU@chru-lille.fr (J.P.); Pierre.COLLINET@chru-lille.fr (P.C.); 2 METRICS: Évaluation des Technologies de Santé et des Pratiques Médicales, University Lille, CHU Lille, ULR 2694, 59000 Lille, France; apierachechr@gmail.com (A.P.); nassima.ramdane.chrulille@gmail.com (N.R.); 3 Department of Gynecological and Breast Surgery and Oncology, Pitié-Salpêtrière University Hospital, Assistance Publique des Hôpitaux de Paris (AP-HP), Sorbonne University, Institut Universitaire de Cancérologie (IUC), 75020 Paris, France; geoffroy.canlorbe@aphp.fr; 4 Gynecological Surgery Service, CHU Lyon-Sud, Pierre-Bénite, 69000 Lyon, France; pierre-adrien.bolze@chu-lyon.fr; 5 Department of Gynecologic and Breast Surgery, Groupe Hospitalier Diaconesses Croix Saint-Simon, 75020 Paris, France; MBallester@hopital-dcss.org; 6 Department of Gynaecology and Obstetrics, Tenon University Hospital, Assistance Publique des Hôpitaux de Paris (AP-HP), University Pierre and Marie Curie, Paris 6, 4 rue de la Chine, 75020 Paris, France; sofiane.bendifallah@yahoo.fr (S.B.); cyril.touboul@gmail.com (C.T.); 7 Department of Obstetrics and Gynaecology, Centre Hospitalier Régional Universitaire de Tours, Hôpital Bretonneau, 2 Boulevard Tonnellé, 37000 Tours, France; l.ouldamer@chu-tours.fr; 8 Department of Obstetrics and Gynecology, Poissy-St Germain Hospital, 10 Rue du Champ Gaillard, 78300 Poissy, France; cyrillehuchon@yahoo.fr; 9 Service de Gynécologie, CHU de Rennes, Université de Rennes 1, 2 Rue Henri le Guilloux, 35033 Rennes, France; Vincent.LAVOUE@chu-rennes.fr; 10 Department of Gynecology and Obstetrics, Centre Hospitalier Intercommunal, Université de Médecine Paris Est Créteil (Paris XII), 94000 Créteil, France; yohann.dabi@gmail.com; 11 Department of Gynecology and Obstetrics, CHU, 67000 Strasbourg, France; cherif.akladios@gmail.com; 12 Department of Surgical Oncology, Georges-François Leclerc Cancer Center, 21000 Dijon, France; ccoutant@cgfl.fr; 13 Department of Obstetrics and Gynaecology, Institute Alix de Champagne University Hospital, 51100 Reims, France; emilie_raimond@hotmail.com; 14 Department of Obstetrics, Gynecology and Reproductive Medicine, CH Jean Verdier, Assistance Publique-Hôpitaux de Paris (AP-HP), av du 14 juillet, 93140 Bondy, France; alex.bricou@gmail.com

**Keywords:** early stage ovarian cancer, minimally invasive surgery, laparoscopic staging, overall survival

## Abstract

Background and objective: according to the latest ESMO−ESGO recommendations, laparotomy is the standard surgical approach to treat and stage patients with presumed early stage epithelial ovarian cancer (EOC). A few studies have investigated the efficacy and the safety of laparoscopy for the staging of early stage EOC, and this question is still in the center of debates. Recurrence-free survival (RFS) and overall survival (OS) benefits of the minimally invasive surgery (MIS) have still to be specified. The aim of this multicenter and retrospective study is to assess the survival outcomes of laparoscopic staging in comparison with laparotomic staging for patients presenting with an early stage EOC. Methods: data of patients with early stage EOC (FIGO I-IIA) who underwent primary surgery between 2000 and 2018 were extracted from the FRANCOGYN database. OS and RFS of these two groups, constituted according to the surgical route, were compared using Log rank test. Results: of the 144 patients included, 107 patients underwent laparotomy and 37 underwent laparoscopy for a staging purpose. The median follow-up was 36.0 months (18.0 to 58.0). For the laparoscopy and the laparotomy group, the median follow-up period was 24 (11.0 to 50.0) and 42.0 (24.0 to 66.0) months, respectively, (*p* < 0.001). Tumor recurrence occurred in 33 (23%) patients: 2 (5.4%) in the laparoscopy group and 31 (29%) in the laparotomy group (*p* = 0.08). The OS rate at 5 years was 97.3% after laparoscopy and 79.8% after laparotomy (*p* = 0.19). Conclusions: there is no difference associated with the laparoscopic approach for the staging of early stage EOC on RFS and OS in comparison with laparotomy. MIS may be proposed as a safe and adequate alternative to laparotomy when performed by well-trained surgeons.

## 1. Introduction

Epithelial ovarian cancer (EOC) is the eighth most common female cancer affecting women in developed countries and strikes one in 70 women [1]. The prognosis remains poor with a five-year overall survival (OS) rate ranging to 43%, all disease stages [1]. It represents the fourth leading cause of death from cancer among women [1]. Although the prognosis of advanced stage EOC (FIGO IIB−IV) is guarded, the prognosis of early stage (FIGO I−IIA) seems good with a five-year OS rate ranging from 80 to 90% [2,3,4]. The standard treatment for early stage EOC consists of total hysterectomy, bilateral salpingo-oophorectomy, omentectomy, appendectomy (when indicated depending on histology), peritoneal cytology, multiple intra-abdominal biopsies and pelvic/para-aortic lymphadenectomy (except for stage I expansive mucinous tumor) by laparotomy followed in most cases by adjuvant platinum-based chemotherapy [1]. The purpose of this surgical management is to assess the final stage of disease and adapt adjuvant therapies and follow-up with minimum perioperative morbidity−mortality. Thus, the performance of this surgical staging is decisive for the prognosis.

Since the rise of minimally invasive surgery (MIS) in the 1990s, the benefits of laparoscopy compared to laparotomy have been clearly recognized: lower postoperative pain, faster recovery of normal bowel function, shorter hospitalization, lower estimated blood depletion, aesthetic scars and more. The introduction of laparoscopy in gynecological cancers has shown the same benefits with similar oncologic outcomes [5,6,7,8]. Weber et al. have also reported others benefits including reduction in the average delay before chemotherapy [8].

Although laparoscopy appears to be an attractive option in the surgical management of early stage EOC, it must provide high-quality staging without compromising efficiency or safety. Thus, the role of MIS in the treatment of early stage EOC is still a matter of debate and there is currently no consensus concerning the surgical route for the staging of early stage EOC. Whereas many studies indicated that laparoscopic staging appeared to be feasible without compromising survival [4,8,9,10,11,12], the Cochrane database review [13] published in 2016 has not provided high-quality evidence to recommend laparoscopy as routine clinical practice. Recently, in the 2019 ESMO−ESGO guidelines, the consensus reports that minimally invasive surgery can be carried out for restaging with a level of evidence IV and a strength of recommendation B. However, laparotomy remains the standard surgical approach to treat and stage patients with apparent early stage EOC with a level of evidence V and a strength of recommendation A [14]. In order to provide reliable information on the long-term efficiency and safety, more studies need to be conducted.

The aim of this multicenter and retrospective study was to assess recurrence-free survival (RFS) and OS in the case of laparoscopic staging in comparison with laparotomic staging for patients presenting an early stage EOC.

## 2. Material and Methods

### 2.1. Study Population

Data of all patients who received primary surgery between 2000 and 2018 were extracted from eleven institutions with maintained EOC database.

The study was approved by the ethics committee of the National College of French Gynecologists and Obstetricians (CEROG 2020-GYN-0302) and patients were duly informed about the study as required by French law.

Clinical and pathological variables included patient age, hormonal status, comorbidities (body mass index (BMI), prior abdominal/pelvic surgery, familial history of gynecological cancer), final international Federation of Gynecology and Obstetrics (FIGO) stage, final pathological analysis (histological type and grade) and adjuvant treatment.

All patients included in this study were early stage EOC. Early stage EOC was defined by stage FIGO I−IIA according to FIGO 2014 classification [15] which means cancer was confined to the ovaries with possible cancer cells in the intra-abdominal fluid secretion or extended to the uterus/the fallopian tubes without cancer cells in the abdominal cavity. Patients were excluded postoperatively if pathological examination confirmed beyond stage IIA ovarian malignancy. All histologic subtypes of the epithelial malignant ovarian tumors were included. Patients with borderline, germ cells and stromal tumors were excluded.

### 2.2. Treatment and Follow-Up

Categorical variables were expressed as numbers (percentage). Patients underwent primary surgical treatment including at least one salpingo-oophorectomy, peritoneal cytology, multiple intra-abdominal biopsies, omentectomy, appendectomy (depending on histology) and pelvic/para-aortic lymphadenectomy. Hysterectomy was performed in the absence of fertility sparing surgery. The entire staging may have been performed in one or two procedures as is frequent in the case of early-stage EOC (for example in the case of an incidental discovery of an EOC on an adnexectomy specimen harvested by laparoscopy). Then, the laparoscopy group included patients who underwent laparoscopy for both diagnosis and staging procedures if two procedures were required and the laparotomy group included patients who underwent laparotomy for both procedures, or only for the staging purpose. As reported in the latest French recommendations [1], there is no selection criteria for performing laparoscopy rather than laparotomy and the choice of the approach was at the discretion of the local team. Chemotherapy was used when indicated. The type of chemotherapy corresponded to carboplatin and paclitaxel. The follow-up was clinical and radiological. Imaging investigations, such as pelvic ultrasound, abdomino-pelvic CT scan, positron emission tomography (PET), were carried out if they were clinically indicated. The frequency of clinical follow-up depended on the practices of each center and was close to the European guidelines which recommend a medical appointment every four months in the first two years, every six months during the three following years and every year thereafter.

At the end of the study, the patterns of recurrence and OS were analyzed. The recurrence was defined by clinical sign, rise of the tumor marker CA-125 or on imaging studies. RFS was defined as the time from admission to hospital for surgery to recurrence. OS was defined as the time from admission to hospital for surgery to death or the last follow-up.

### 2.3. Statistical Analysis

Continuous variables were expressed as means (±standard deviation, SD) in the case of normal distribution or medians (interquartile range) otherwise. Normality of distribution was assessed using histograms and the Shapiro−Wilk test. OS and RFS were estimated using the Kaplan−Meier method.

Comparison of patients’ characteristics and surgical outcomes between the two study groups (laparoscopy and laparotomy) were done using Chi-square tests (or Fisher’ exact tests when expected cell frequency was <5) for categorical variables and Student t or Mann−Whitney U tests (regarding the normality of distributions) for continuous variables.

We compared RFS and OS by using Log rank test. Moreover, RFS and OS was evaluated after adjustment for histological grade and for FIGO stage by using Cox’s regression models and derived adjusted hazards ratio (HRs) as effect size measures, with their 95% confidence intervals (CIs). We assessed the proportional hazard assumption using Schoenfeld residuals plots [16].

To handle the missing values on histological grade, we used multiple imputation with a regression switching approach (chained equations with m = 10). Imputation procedure was performed under the missing at random assumption using all variables listed in Table 1 (including study group) with a predictive mean matching method for continuous variables and logistic regression model for categorical variables. We combined effect sizes from each imputed dataset using Rubin’s rules [17].

Statistical testing was done at the two-tailed α level of 0.05. Data were analyzed using SAS software package, release 9.4 (SAS Institute, Cary, NC, USA).

## 3. Results

### 3.1. Study Population

**Figure 1:**
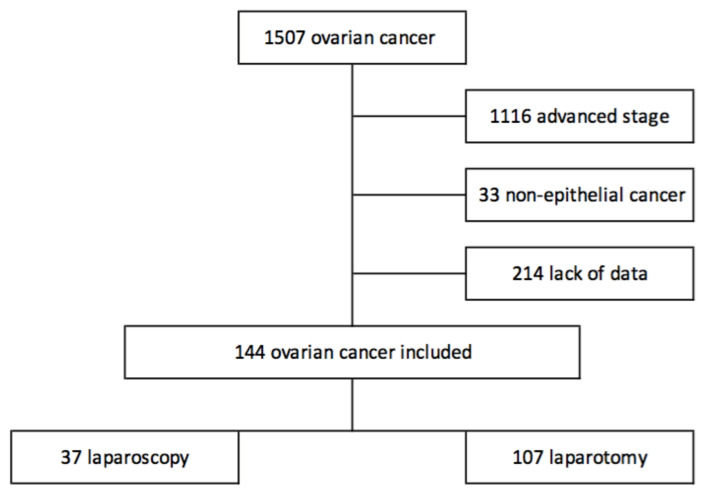
Flowchart.

The 144 patients meeting the inclusion criteria were analyzed. There were 107 patients who underwent laparotomy and 37 who underwent laparoscopy for a staging purpose. The flowchart of the study is reported in Figure 1.

### 3.2. Characteristics

Patient characteristics are shown in Table 1. No difference in baseline characteristics was observed between the two groups, including age, BMI, hormonal status, family history of gynecological cancer, prior abdominal surgery, surgical FIGO stage, and histologic type. In the laparotomy group, a lower proportion of patients had high histologic grade than patients in the laparoscopy group (46.7% vs. 77.3%, *p* = 0.014).

### 3.3. Patients’ Management

The surgical outcomes are shown in Table 2. No difference was found regarding the achievement of lymphadenectomy between the two groups. The median number of pelvic nodes removed was 12 (6 to 18) in the laparoscopy group and 7 (0 to 13) in the laparotomy group (*p* = 0.026). There was no significant difference for para-aortic node removed (*p* = 0.27). There was no significant difference in the incidence of intra- and postoperative complications between the two groups. In the laparoscopy group, the following postoperative complications were reported: 2 vaginal disunions, 2 digestive occlusions, 1 unspecified sepsis, 1 unspecified neurological deficit, 1 hemoperitoneum. In the laparotomy group, the following postoperative complications were reported: 1 vaginal disunion, 2 lymphoceles, 3 fistulas without precision, 1 eventration, 1 digestive occlusion, 1 intestine valve, 1 hemoperitoneum, 1 ischemic stroke, 1 episode of confusion. There were 4 tumor ruptures in the laparotomy group and 0 in the laparoscopy group. Adjuvant chemotherapy was required for 91.9% patients in the laparoscopy group and for 89.7% patients in the laparotomy group (*p* = 0.70). The median number of adjuvant cures in the laparoscopy group and in the laparotomy group was 6 (0.0 to 6.0) and 6 (5.0 to 6.0), respectively, with no significant difference (*p* = 0.19).

### 3.4. Overall and Recurrence-Free Survival

The overall median follow-up period was 36.0 months (18.0 to 58.0). For the laparoscopy and laparotomy group, the median follow-up period was 24.0 (11.0 to 50.0) and 42.0 (24.0 to 66.0) months, respectively (*p* < 0.001).

**Figure 2:**
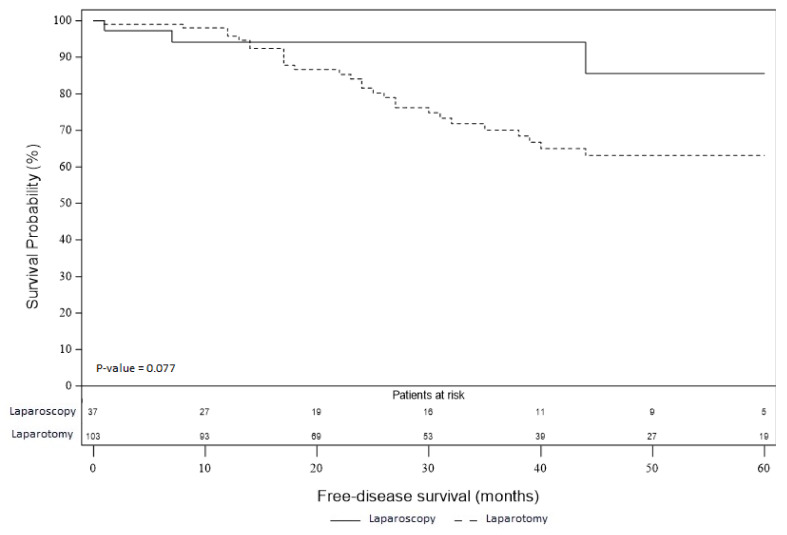
Recurrence-free survival of the laparoscopy and laparotomy groups.

**Figure 3:**
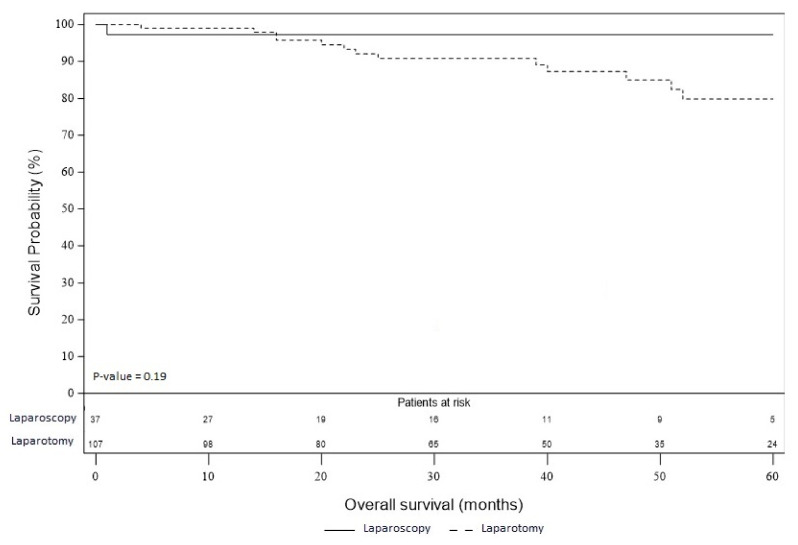
Overall survival of the laparoscopy and laparotomy groups.

RFS and OS are presented in Figure 2 and Figure 3. Thirty-one patients developed tumor recurrence in the laparotomy group and two patients in the laparoscopy group. In the laparotomy group, there were 16 peritoneal recurrences, 4 lymph node recurrences, 9 distant metastases and this data was missing for two patients. In the laparoscopy group, one patient developed peritoneal recurrence, and distant metastases were recorded in one patient. RFS was better in the laparoscopy group than in the laparotomy group, however, the difference did not reach the significance level (*p* = 0.08). This result was similar after adjustment on histological grade with an HR of 0.36 (95% CI, 0.11 to 1.19; *p* = 0.095), as well as after adjustment on FIGO stage with an HR of 0.39 (95% CI, 0.12 to 1.30; *p* = 0.12). Seventeen patients died in the laparotomy group: four were attributed to the evolution of the disease, two during digestive complications (occlusive syndrome, digestive fistula), two died from postoperative complications related to patient comorbidities (respiratory distress and multivisceral failure), and we did not have the information for the last nine patients. In the laparoscopy group, one patient died because of intracranial hemorrhage from chemotherapy-induced thrombocytopenia. Although there was a difference of about 17% in favor of laparoscopy, there was no significant difference in OS rate between the two groups (*p* = 0.19). OS rate at 5 years in the laparoscopic group was 97.3% and 79.8% in the laparotomy group. This result was similar after adjustment on histological grade with an HR of 0.28 (95% CI, 0.04 to 2.13; *p* = 0.22). In the same way, OS at 5 years did not reach the significance level after adjustment on FIGO stage with an HR of 0.30 (95% CI, 0.04 to 2.34; *p* = 0.25).

## 4. Discussion

In our study concerning the surgical staging of early stage EOC, there is no difference associated with the laparoscopic approach on survival outcomes in comparison with laparotomy. Indeed, no difference was found in RFS and OS after laparotomic or laparoscopic staging.

These results are similar to other trials previously published [9,12,13,18,19,20,21]. However, the safety of the MIS remains controversial. The ESMO−ESGO, the UK and the NCCN guidelines still consider laparotomy as the chosen surgical procedure for this pathology whereas German and French recommendations included the option of laparoscopy [4,13,14].

The greatest concern for the use of laparoscopy is the high-risk of intraoperative tumor rupture. However, this risk has also been described with laparotomic staging. In our study, 4 tumor ruptures were observed in the laparotomy group and none in the laparoscopy group. Among them, there was no recurrence or death noticed for three patients with follow-up durations of 31, 44 and 55 months. The other one died after 92 months of follow-up. Unfortunately, we had no information on the size of the tumor, which may have influenced the chosen surgical route. It is difficult to establish a causal relationship between the occurrence of tumor rupture and its influence on survival data due to the small number of patients. Many studies have reported that the incidence of tumor rupture was similar between the laparoscopy and laparotomy group and ranges from 11.4 to 30.3% [10]. Vergote et al., in the largest retrospective, multicenter study involving >1500 patients, reported that rupture during surgery would appear to be associated with a poor prognosis for patients with stage I EOC [22]. In the same way, in the metanalysis of Bentivegna et al., it has been reported that stages 1C2−3 have a higher risk of recurrence [23] Actually, the clinical significance remains uncertain [24]. A retrospective review of 394 patients found no difference in survival outcomes [25]. Similarly, Suh et al. reported that surgical spillage of tumor cells did not appear to have a negative effect on survival [26]. Although the association between intraoperative rupture and survival outcomes are not obvious, we must try to minimize the risk of tumor contamination in the abdominal cavity. All efforts should be made by using a laparoscopic bag and controlled aspiration. Moreover, the risk of tumor rupture increases with the size of the tumor and it could be an argument to choose laparotomy more than laparoscopy for the surgical staging. To reduce the intraperitoneal spillage of tumors cells Lu et al. performed irrigation of the intraperitoneal cavity using distilled water and cisplatin at the end of the surgical procedure [2].

One of the other controversial issues is port-site metastases. The rate of occurrence of port-site metastases is 1–2% for patients undergoing laparoscopic surgery for malignancies [27]. In our study, there were no port-site metastasis or abdominal wall metastasis reported. Mechanisms remain unclear (hematogenous spread, direct wound contamination, the effect of carbon-dioxide pneumoperitoneum, local immune reactions, the surgical technique used…) [2]. Nezhat et al. found no significant difference in the rate of abdominal wall metastases after laparotomy in comparison with the rate of port-site metastases after laparoscopy for EOC [11]. Most of the reported cases occurred at the same time as other sites of metastases [27]. These results suggest that port-site metastases and abdominal wall metastases are associated with advanced diseases, independently of the initial surgical route. The use of a laparoscopic bag, closure of the trocar site at the end of the procedure can protect of the risk of trocar site metastases [11]. To decrease the incidence of port-site metastases, Lu et al. removed the tumor through the vagina, when hysterectomy was performed, by placing a pipe in the vaginal canal to avoid contact with the vaginal wall [2].

Since the 1990s, MIS had proved its benefits compared to open surgery. Bogani et al. showed in a meta-analysis of eleven retrospective studies including a total of 3065 patients that MIS was associated with statistically better operative outcomes in comparison with laparotomy [28]. Minig et al. reported significant reduction of estimated blood loss, blood transfusion, length of hospitalization time in comparison to surgical staging performed by laparotomy [18]. Zhang et al. suggested that laparoscopy preserved patients’ cellular immunity and we know that immune defenses are essential for combating the disease [29]. Laparoscopic staging can provide better visualization of the peritoneal surface thanks to the optical magnification compared to laparotomy [2,8,29]. However, total inspection of the peritoneum and critical areas (as hepatophrenic ligament, lesser omental sac, porta hepatis, splenophrenic ligament, hidden space in the folded intestine, implants of the mesentery) can be limited with laparoscopy. It could resulted in failure of upstaging and inadequate adjuvant treatment [19]. But no differences in the proportion of patients upstaged between the laparoscopy and laparotomy group have been found [9,21]. Furthermore, the laparotomy allows for fine tactile assessment of the extent of the disease, which is not possible during the laparoscopic staging [2,29]. To compensate for the lack of tactile sensation, the use of hand-assisted laparoscopic surgery (HALS) could be proposed [2]. HALS consists of traditional laparoscopy combined with a small periumbilical or suprapubic vertical midline incision equal to surgeon’s glove size [30]. The surgeon introduces his hand intraperitoneally, and with the assistance of the laparoscope, he has the ability to visualize and palpate all peritoneal surfaces and retroperitoneal structures.

Although the rate of patients who have benefited of pelvic and para-aortic lymphadenectomy seems to be low, no difference was found between the two groups. Comparing the number of lymph nodes removed may be another way to assess the quality of the surgical staging after laparoscopy or laparotomy. Although the median or mean number of lymph nodes can vary significantly, ranging from 13 to 22 for pelvic node dissection and 7 to 34 for para-aortic node dissection, the review of the literature does not find any difference between the two surgical approaches [2,18,24]. In the present study, laparoscopy was not worse in terms of lymph nodes removed and even better concerning the pelvic lymph nodes.

Moreover, the meta-analysis of Bogani et al. reported that laparoscopic staging was associated with a reduction in time to the beginning of chemotherapy than open surgery [28]. According to the latest French recommendations, the chemotherapy should start within 6 weeks of the surgery [1]. Laparoscopy, allowing better intraoperative and postoperative outcomes, could reduce the delay between surgery and chemotherapy and thus improve survival outcomes.

**Figure 4:**
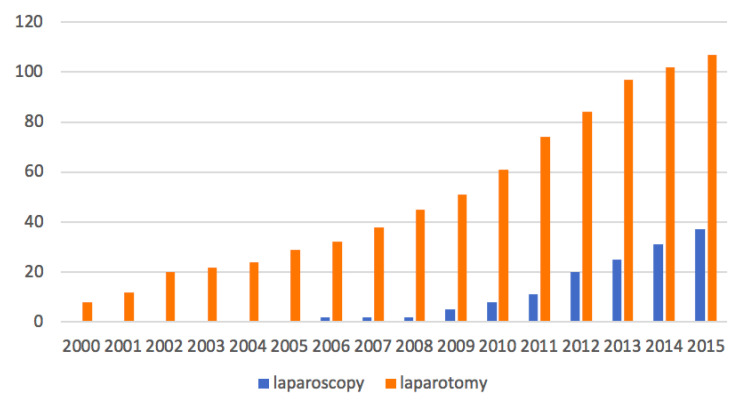
Distribution of patients according to the surgical route over time.

The strengths of our study are the consistency and homogeneity of our patient population and the relatively long follow-up compared to other trials previously published (Table 3). Our study was limited because of its design. The retrospective nature of the database might be associated with a possible selection bias and under-recorded clinical data. Ovarian cancer is mainly diagnosed at an advanced stage; we therefore had to exclude 1116 patients for advanced stage (within the 1509 patients reported in the database). Thirty-three other patients had been excluded because they had a nonepithelial histological type. We also had to exclude a significant number of patients (*n* = 214), including patients with early stage EOC, in order to have the most precise and complete data. Of these 214 patients, 119 were excluded due to lack of information on survival. The 95 patients left were excluded for missing histology or precise disease stage information. This has led to the study of groups with smaller numbers of patients that may have resulted in the lack of detecting a statistical difference. There were more numerous cases in the laparotomy group. This result was expected since laparotomy remains the gold standard in the staging of EOC. Moreover, the wider use of laparoscopy in EOC is more recent. The greater proportion of patients with high histologic grade in the laparotomy group is difficult to interpret. Fortunately, the difference in OS and RFS between the two groups did not reach the significant level after adjustment on histological grade. Another bias is the median follow-up, which differed significantly between the two groups. Indeed, we cannot exclude that a longer follow-up in the laparoscopy group might reveal a significantly negative impact of this surgical route on survival outcomes. However, literature review shows similar outcomes (Table 3). Lu et al. with the longest follow-up of 82 months for the two groups, reported OS rates of 92.9% in the laparoscopy group and 90% in the laparotomy group (*p* = 0.35), and RFS rates of 87% for both groups with time to recurrence above 20 months postoperatively [2]. Gadducci et al. in a retrospective study of 224 patients found a median time to recurrence of 29 months (range, 5–112 months) [31]. The difference of follow-up durations between the two groups could be explained by the fact that the proportion of patients operated on more recently is greater in the laparoscopy group than in the laparotomy group. Indeed, in the laparoscopy group, 78.4% of patients were operated on after 2010 while in the laparotomy group, 52% of patients were operated on after 2010. Thus, this difference in inclusion over time results in shorter inclusion time for the laparoscopy group, which reduced the number of cases included in this group and explain a shorter period of follow-up. Concerning the laparotomy group, the fact that 52% of patients were included after 2010 with the latest inclusion in 2015 explains the follow-up of 42 months by reducing the inclusive period. The distribution of patients according to the surgical approach over time is represented in Figure 4. The continuation of this study over time may improve the duration of follow-up and may be the subject of another study in a few years’ time. Finally, it would have been interesting to provide an additional analysis of complications and survival for patients who underwent one-step versus two-step procedures for the complete surgical staging of the cancer. Unfortunately, it was not possible for us to realize it in a precise way because of the data available in our database.

We caution that the present findings were derived from an observational, nonrandomized design with limited sample size and despite any statistical models to address imbalanced data regarding study groups, we could not exclude confusion and selection bias as any observational study. Regarding the differences in follow-up between the two groups, we believe that there is no informative censoring reason which could strongly bias the difference between the two groups.

Although laparoscopy has become essential in the management of gynecological cancers, the results of the LACC trial (Laparoscopic Approach to Cervical Cancer) bring us to go back forward to laparotomy for management of early stage cervical cancer [32,33]. However, the scientific community still does not understand well the mechanisms which could explain a greater rate of recurrence and death with laparoscopy in comparison with laparotomy for cervical cancer management [34]. There is probably a place for MIS in the surgical management of cervical cancer (without uterine manipulator and primary vaginal closure) [35] but this place needs to be validated by further investigations.

While we must remain vigilant about conclusions regarding MIS to staging early stage EOC, the data from this retrospective study encourage further prospective research in this area. Nevertheless, it could be difficult to recruit enough patients for such a study. EOC is uncommon and is often detected at an advanced stage. Therefore, the comparison between the two surgical procedures is relevant even through only indirect evidence such as retrospective analysis. More studies with a larger number of patients in the laparoscopy group, high quality of follow-up, longer and equal in the two groups, need to be conducted.

## 5. Conclusions

For the surgical staging of early stage EOC, laparoscopy has shown in this study similar oncologic outcomes in comparison to laparotomy. The benefits of MIS are that it may improve the comfort of patients and improve delays to begin adjuvant treatment. In this way, laparoscopic staging of early EOC may be an adequate and a safe alternative to laparotomy for selected patients when performed by well-trained surgeons. Prospective studies are still awaited to confirm these findings.

## Figures and Tables

**Table 1:** Patient characteristics.

Characteristic	Laparoscopic (*n* = 37)	Laparotomy (*n* = 107)	*P* Value
Age (y)	56.3 (±16.8)	56.2 (±14.7)	0.98
BMI (Kg/m^2^) ^1^	23.8 (±5.0)	25.4(±5.0)	0.13
Hormonal status			0.56
Menopausal	31/36 (86.1)	93/104 (89.4)	
Nonmenopausal	5/36 (13.9)	11/104 (10.6)	
Family history of gynecological cancer	10/34 (29.4)	24/100 (24.0)	0.53
Prior abdominal surgery	13/30 (43.3)	16/41 (39.0)	0.72
Surgical FIGO stage			0.24
IA	18/37 (48.7)	51/107 (47.7)	
IB	1/37 (2.7)	10/107 (9.4)	
IC	12/37 (32.4)	38/107 (35.5)	
IIA	6/37 (16.2)	7/107 (6.5)	
Grade			0.014
high	17/22 (77.3)	28/60 (46.7)	
Low	5/22 (22.7)	32/60 (53.3)	
Histology			0.42
Serous	17/37 (46.0)	35/107 (32.7)	
Mucinous	7/37 (18.9)	18/107 (16.8)	
Endometrioid	8/37 (21.6)	31/107 (29.0)	
Clear cell Mixed	5/37 (13.5) 0/37 (0.0)	18/107 (16.8) 5/107 (4.7)	

BMI = body mass index, FIGO = International Federation of Gynecology and Obstetrics. Values are expressed as mean (±standard deviation) or no./total no. (percentage). ^1^ 19 missing values (17 patients with laparotomy).

**Table 2:** Patients’ management.

Variables	Laparoscopy (*n* = 37)	Laparotomy (*n* = 107)	*p* Value
Pelvic lymphadenectomy	28/37 (75.7)	67/105 (63.8)	0.19
Para-aortic lymphadenectomy	27/37 (73.0)	67/107 (62.6)	0.25
Pelvic node removed	12 (6 to18)	7 (0 to13)	0.026
Para-aortic node removed	14 (0 to23)	9 (0 to20)	0.27
Intra operative complications Tumor rupture Organ damage	3/22 (13.6) 0 3	6/105 (5.7) 4 2	0.19 NA NA
Postoperative complications	7/34 (20.6)	11/99 (11.1)	0.24
Chemotherapy	34/37 (91.9)	96/102 (94.1)	0.70

Values are expressed as no./total no. (percentage) or median (interquartile range). 1: 52 missing values (44 patients with laparotomy), 2: 59 missing values (49 patients with laparotomy) NA = not applicable.

**Table 3:** Literature data.

First Author	Year	Surgical Approach (*n*)	Recurrence *n* (%)	Odds Ratio, 95% CI	Death of Disease *n* (%)	Odds Ratio, 95% CI	Follow-up (Months)
Ditto	2016	MIS (*n* = 50) OPEN (*n* = 50)	7 (14.0) 11 (22.0)	0.79 (0.31–2.01)	2 (4.0) 5 (10.0)	0.87 (0.08–9.19)	49.5 (+/−64) 52.6 (+/−31.7) *p* = 0.01 **
Gallota	2016	MIS (*n* = 60) OPEN (*n* = 120)	5 (8.3) 16 (13.3)	NR * p = 0.651	5 (8.0) 11 (9.0)	NR * p = 0.72	38 (24–48) 38 (24–48)
Lu	2016	MIS (*n* = 42) OPEN (*n* = 50)	5 (13.0) 6 (13.0)	NR * p = NS	3 (7.1) 5 (10.0)	NR * p = 0.63	82 (16–152) 82 (16–152)
Melamed	2016	MIS (*n* = 1096) OPEN (*n* = 1096)	NR NR	Not estimable	55 (5.1) 71 (6.5)	0.77 (0.54–1.09)	28.7 (20.4–38.9) 29.3 (20.6–39
Minig	2016	MIS (*n* = 50) OPEN (*n* = 58)	6 (12.0) 7 (12.0)	0.50 (0.21–1.21)	NR NR	NR * p = 0.42	25.9 (11.2–38.5) 34.3 (28.4–47.8) *p* = 0.004 **
Bogani	2014	MIS (*n* = 35) OPEN (*n* = 32)	4 (11.4) 9 (28.1)	0.33 (0.09–1.20)	2 (5.7) 4 (12.5)	NR * p = 0.26	64 (37–106) 100 (61–278) *p* <0.001 **
Liu ***	2014	MIS (*n* = 35) OPEN (*n* = 40)	3 (8.6) 2 (5.0)	1.78 (0.28–11.33)	1 (2.9) 1 (2.5)	1.15 (0.07–19.05)	NR (36–84)
Koo	2013	MIS (*n* = 24) OPEN (*n* = 53)	2 (8.3) 2 (3.8)	NR * p = 0.59	1 (4.2) 0 (0.0)	NR * *p* = 0.23	31.7 (+/−20.7) 31.1 (+/−19.1)
Lee ***	2011	MIS (*n* = 26) OPEN (*n* = 87)	0 (0.0) 0 (0.0)	Not estimable	0 (0) 3 (3.4)	0.46 (0.02–9.11)	12 (1–42) 25 (1–74)
Park (2) ***	2008	MIS (*n* = 19) OPEN (*n* = 33)	0 (0.0) 0 (0.0)	6.29 (0.28–140.86)	0 (0) 0 (0)	3.55 (0.14–93.01)	17 (2–40) 23 (1–44)
Park (1) ***	2008	MIS (*n* = 17) OPEN (*n* = 19)	2 (12.0) 0 (0.0)	Not estimable	1 (5.9) 0 (0)	Not estimable	19 (5–56) 14 (5–61)
Ghezzi ***	2007	MIS (*n* = 15) OPEN (*n* = 19)	0 (0.0) 4 (7.1)	NR	0 (0.0) 0 (0.0)	NR	16 (4–33) 60 (32–108)

MIS = minimally invasive surgery as laparoscopy; OPEN = open surgery as laparotomy; * Results not expressed as OR but means no significant difference on the survival outcomes (*p* > 0.05). ** Significant difference concerning the follow-up between MIS and OPEN groups. *** Studies lacking data concerning the follow-up.
